# GPLEXUS: enabling genome-scale gene association network reconstruction and analysis for very large-scale expression data

**DOI:** 10.1093/nar/gkt983

**Published:** 2013-10-30

**Authors:** Jun Li, Hairong Wei, Tingsong Liu, Patrick Xuechun Zhao

**Affiliations:** ^1^Plant Biology Division, the Samuel Roberts Noble Foundation, 2510 Sam Noble Parkway, Ardmore, OK 73401, USA and ^2^School of Forest Resources and Environmental Science, Michigan Technological University, 1400 Townsend Drive, Houghton, MI 49931, USA

## Abstract

The accurate construction and interpretation of gene association networks (GANs) is challenging, but crucial, to the understanding of gene function, interaction and cellular behavior at the genome level. Most current state-of-the-art computational methods for genome-wide GAN reconstruction require high-performance computational resources. However, even high-performance computing cannot fully address the complexity involved with constructing GANs from very large-scale expression profile datasets, especially for the organisms with medium to large size of genomes, such as those of most plant species. Here, we present a new approach, GPLEXUS (http://plantgrn.noble.org/GPLEXUS/), which integrates a series of novel algorithms in a parallel-computing environment to construct and analyze genome-wide GANs. GPLEXUS adopts an ultra-fast estimation for pairwise mutual information computing that is similar in accuracy and sensitivity to the Algorithm for the Reconstruction of Accurate Cellular Networks (ARACNE) method and runs ∼1000 times faster. GPLEXUS integrates Markov Clustering Algorithm to effectively identify functional subnetworks. Furthermore, GPLEXUS includes a novel ‘condition-removing’ method to identify the major experimental conditions in which each subnetwork operates from very large-scale gene expression datasets across several experimental conditions, which allows users to annotate the various subnetworks with experiment-specific conditions. We demonstrate GPLEXUS’s capabilities by construing global GANs and analyzing subnetworks related to defense against biotic and abiotic stress, cell cycle growth and division in *Arabidopsis thaliana*.

## INTRODUCTION

The availability of terabyte- and petabyte-sized gene expression datasets in public repositories (1,2) has inspired scientists to use genome-wide reverse genetic approaches to reconstruct gene networks and decipher the interaction between genes. Compared with forward genetics, which usually focuses on individual genes, the reverse-engineering that focuses on genome-wide transcriptome profiles can provide a holistic and comprehensive view of the interactions of an entire network, which may lead to the discovery of novel regulatory relationships that underpin each biological process or trait (3–5). Although the genome-wide reverse-engineering approach has many advantages, several computational challenges exist, particularly for organisms with large genome datasets such as those of plants. The computational complexity grows exponentially as the number of genes increases, and an increase in the number of genes, in turn, demands an accordingly increased sample size to achieve the desired accuracy for network reconstruction.

Among the available gene association network (GAN) algorithms for the reverse engineering of large-scale gene networks (6–9), the gene co-expression network method (6) is a commonly used method to infer potential genetic interactions from gene expression datasets. One problem that is inherent in this co-expression network method is its high false-positive prediction rate, which is due to its inability to distinguish direct gene interactions from large number of indirect interactions. Other methods, such as the Bayesian Network (7) and Gaussian Graphics Model (GGM) (9), can infer the local network structure with high precision (10), but cannot handle genome-wide network construction due to the increased computational complexity that arises from the large number of gene variables (10) and the large sample requirement (11). To date, constructing GANs from very large-scale expression profile datasets, especially for the organisms with medium to large size of genomes, such as those of most plant species, is still a challenging task, which necessitates the development of more effective computational solutions.

Several methods have been developed to infer GANs using mutual information (MI)-based approaches (12,13), including the Algorithm for the Reconstruction of Accurate Cellular Networks (ARACNE) method (12), which can identify both linear and non-linear dependence relationships from large samples. Furthermore, a large number of potential indirect gene–gene interactions can be eliminated using the ARACNE method through the application of data processing inequality (DPI), which is based on information theory (14). The ARACNE method (4), which is now widely used in GAN reconstruction (15,16), achieves better performance than the Bayesian Network method (7). However, this method remains computationally demanding due to the high computational complexity of both the MI estimation, which uses Gaussian kernel-based methods, and the DPI processing, which is applied to a large number of edges. Most GANs that are deciphered by this method have therefore adopted compromise strategies that either reduce the number of genes included in the networks or use a relatively small number of gene expression profiles (4,17). Even with these compromises, ARACNE requires >100 h to calculate the MI of all gene pairs for the human B-cell expression dataset (18), which only contains ∼10 000 genes across 336 samples, on a typical modern server with 32 CPU cores and 128-GB RAM. For this reason, fast MI estimation methods (18,19) have been developed. However, these methods usually shorten the computational time at the cost of memory consumption, which often reduces the ability to process massive datasets. Meanwhile, the application of a DPI filter to remove potentially false interactive edges is also a time-consuming process that cannot be easily reduced. Therefore, the development of ultra-fast methods to enable the construction of genome-wide association networks, particularly for plant species with large genomes, is imperative for the field of genomics.

Another open question in genome-wide GAN analysis in plants is how to effectively identify functional subnetworks that control specific cellular processes, such as development or response to environmental cues. Sessile plants are subjected to constant biotic and abiotic stressors, and the study of the interaction of plants with the environment can lead to adaptation-ameliorated plant varieties. For example, well-adapted plants have been selected during crop domestication to meet the increased needs of human beings. Evidence has shown that the major evolutionary changes in plant adaptation (20,21) can be linked to the modification of higher hierarchical regulatory relationships (22,23). Therefore, the link between gene–gene associated pairs within a subnetwork and the experimental condition under which the pair was observed is an important subject in modern plant genomics and systems biology.

Here, we present a new integrated approach for high-performance GAN construction and analysis from large-scale data using an ultra-fast MI estimation that uses a Spearman correlation-based transformation for pairwise MI computing and removes potentially false interaction edges on the basis of DPI. We further reduce the computational time by implementing integrated GAN construction and network analysis algorithms on a custom-built parallel-computing Linux cluster platform, BioGrid, which accelerates the total computational process in almost linear proportion to the number of CPU cores that are used. We demonstrate GPLEXUS’s capability by analyzing large-scale *Arabidopsis thaliana* gene expression datasets that have been pooled from multiple experimental conditions to first build a genome-wide GAN and then decompose this GAN into subnetworks. Moreover, we have developed a novel function to identify major experimental conditions that contribute to the MI of gene–gene interactions in the constructed networks, which allows users to link each gene–gene association or subnetwork to a specific experimental condition to learn under which condition these gene–gene associations may operate.

To promote and facilitate the use of this platform to perform GAN analyses for organisms with large genomes and a large number of genes, we have provided a user-friendly online platform (http://plantgrn.noble.org/GPLEXUS) that allows users to upload their expression datasets and perform GAN and gene set enrichment analysis. To the best of our knowledge, this is the first web-based platform that is able to construct and analyze genome-scale GANs from massive genomic datasets.

## MATERIALS AND METHODS

### Datasets used for method evaluation

Four compendium datasets were downloaded from public domains and compiled to evaluate the performance of GPLEXUS and other methods (Table 1). The first three datasets were downloaded from ArrayExpress (1). Dataset I comprises gene expression profiles of 313 microarray hybridizations for *Escherichia coli*. Dataset II comprises gene expression profiles of 1848 microarray hybridizations for *A. thaliana*. Dataset III comprises 768 microarray hybridizations from multiple tissues for *Glycine max*. Dataset IV comprises human glioblastoma gene expression profiles, which were downloaded from the TCGA Data Portal (Level 1, Affymetrix HT Human Genome U133 Array) (24). All of these datasets were normalized using the Robust Multiarray Averaging (RMA) method (25) and were used to evaluate and compare the performance of GPLEXUS with other methods. All four datasets are available for download on the GPLEXUS Web site (*http://plantgrn.noble.org/GPLEXUS/dataset.jsp*).

**Table 1.** Comparison of the performance of several integrated methods on four compendium datasets

Species/cell line	Number of arrays	Number of probe sets	Runtime (minutes)				
			M1	M2	M3	M4	M5
*Escherichia coli*	313 (Dataset I)	15 552	12 640	1034	40	12	7
*Arabidopsis thaliana*	1848 (Dataset II)	22 810	^a^	^a^	5800	51	34
*Glycine max*	738 (Dataset III)	66 190	^a^	^a^	12 000	88	42
Human glioblastoma	547 (Dataset IV)	22 277	30 640	12 260	180	18	12

M1: Original ARACNE method; M2: Spearman-based MI estimation with integrated DPI analysis; M3: Parallel implementation of the original ARACNE method deployed on our BioGrid system; M4: Parallel implementation of the B-Spline-based MI estimation with integrated DPI analysis deployed on our BioGrid system; M5: Parallel implementation of the Spearman-based MI estimation with integrated DPI analysis deployed on our BioGrid system.

^a^Computationally infeasible.

### Fast MI estimation

The ARACNE method had successfully applied the properties of MI and DPI, which are based on information theory (14), to GAN reconstruction (4). Mounting evidence has also demonstrated the effectiveness of ARACNE to build valid and explainable gene networks (26–28). The network construction from microarrays of plant species, which usually contain a very large number of genes, is often time-consuming and sometimes computationally infeasible. To reduce the computational complexity of the MI estimation, we have developed a fast MI estimation method that is based on the Spearman correlation-based transformation formula for MI computing. One report (29) has proven that the Pearson’s correlation and MI are related when the sample data follow a normal distribution. For this case, the value of the MI can be calculated as:
(1)
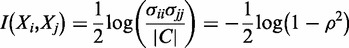

where 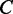 is the determinant of the covariance matrix, 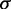 is the standard deviation of the gene expression dataset and 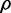 is the Pearson’s correlation. The Spearman correlation coefficient is a special case of the Pearson’s correlation in which the data are converted to ranks before estimating the coefficient. The MI computed by Spearman correlation-based transformation can capture both linear and non-linear relationships. Furthermore, the use of the Spearman correlation-based transformation also enables the application of DPI to remove potential indirect genetic relationships from the constructed network. Both the Spearman correlation-based transformation-based MI estimation method and the Gaussian kernel-based MI estimation method used by the original ARACEN algorithm have been integrated into GPLEXUS. The Spearman correlation-based transformation method is much faster than the ARACNE method; the MI computational complexity of GPLEXUS is 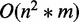, whereas the MI computational complexity of ARACNE is 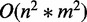, where *n* is the number of microarray probe-sets/genes and *m* is the number of microarray hybridizations/samples.

### Ultrafast MI computing and DPI processing via parallel computing

We implemented the integrated algorithms with parallel programming techniques in an efficient C++ and Java computing languages and deployed the GPLEXUS analysis pipelines on an in-house Linux cluster called BioGrid, which currently consists of >700 CPU cores to achieve a high-performance computing capacity.

When a user submits an analysis job through the GPLEXUS online web server, the master node of the BioGrid system first transfers the datasets to slave computing nodes in the Linux cluster. Next, the master node remotely calls to execute the analysis pipelines and monitors the analysis progress in these computing nodes. The master node collects the analysis outputs when all of the distributed jobs have been completed. This procedure is iterated twice to first complete the MI estimation and then to remove indirect edges by DPI analysis. The initial network construction can be further refined by iteratively re-running the analysis pipelines with more stringent parameters. By default, GPLEXUS estimates and chooses the MI of the 10th percentile of N-ordered values (arranged from the largest to the smallest) as the default MI threshold, and a *P*-value is assigned to this MI threshold by random shuffling of the expression of these gene–gene pairs across the various microarray profiles. The computational complexity of the Spearman correlation-based transformation and the DPI processing is further reduced through the parallel-computing implementation to 
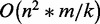
 and 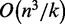, respectively, where *n* is the number of microarray probe-sets/genes, *m* is the number of microarray hybridizations/samples and *k* is the number of CPU cores in the BioGrid system.

### A ‘condition-removing’ approach to identify experiment-specific conditions for gene-gene associations

To infer the potential experimental conditions under which gene–gene interactions/regulations may occur, we have developed a ‘condition-removing’ approach to infer the experimental conditions of the microarray. The principle of the approach supposes that if a regulated relationship occurs under a specific experimental condition, then the MI value for the gene pair would be reduced if this experimental condition was removed from all of the microarrays. A larger decrease in the MI value indicates a higher likelihood that the gene–gene pair is regulated or interacts under this condition. Therefore, a series of MI values can be estimated for every gene–gene pair by removing experimental conditions one-by-one. Because all of the MI values for one gene pair would follow a normal distribution (in general, the MI values could be estimated accurately from >100 microarray chips), a one-side *z*-test could be applied to identify the experimental conditions that lead to significant reductions in the MI value (default *P*-value ≤10^−^^4^). All of these identified conditions would cover the range of biological conditions in which each possible gene–gene interaction exists. The computational complexity of this method is 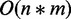. This analysis cannot be performed easily using a single server on a dataset that contains 10 000 identified gene–gene links and >1000 chips. However, the ultra-fast Spearman correlation-based MI estimation on the GPLEXUS high-performance computing platform can perform this analysis in less than a few minutes.

### Subnetwork discovery by Markov clustering analysis

In general, biological networks are ‘scale-free’ (30). Therefore, a network clustering method that simulates a random walk, such as the Markov Clustering Algorithm (MCL) (31), can identify actual or potential functional subnetworks with a high degree of accuracy. We integrated this method into our GPLEXUS platform to partition the networks into functional subnetworks.

### GPLEXUS online

We provide a user-friendly version of GPLEXUS, called GPLEXUS Online (http://plantgrn.noble.org/GPLEXUS), as a public and freely available web server that allows users to upload their expression datasets, perform GAN analysis and download the analysis results. The analysis flow of GPLEXUS Online is illustrated in Supplementary Figure S1.

#### Uploading expression data

The input data should consist of normalized expression data in a tab-delimited text format. The first column of the data is the gene identifier, such as the GeneChip Probe-set ID or gene name, and the other columns are the expression values. The first row refers to the chip name so that GPLEXUS can identify chip names during the experimental condition identification analysis.

#### MI estimation algorithm selection

Two methods are provided in GPLEXUS to estimate the MI: the Spearman correlation-based transformation MI estimation method (this is the default method) and the Gaussian-kernel-based MI estimation method that was used in the original ARACNE method.

#### Iterative GAN construction and refinement

When the data are submitted for analysis, GPLEXUS automatically performs a parameter estimation and constructs ‘coarse’ networks with less stringent parameters. The MI threshold is set as a value that includes the top 10% of gene–gene pairs and a DPI tolerance of 1.0. The coarse networks can be viewed as original relevance networks (13) that have no indirect edges removed. The user may further adjust and filter the initial constructed networks by applying more stringent parameter settings, i.e. by adjusting both the MI threshold and DPI tolerance values. A high-precision network can be reached via a ‘coarse-to-fine’ refining process as described.

#### Functional subnetwork discovery

GPLEXUS automatically performs an MCL clustering analysis for functional subnetwork discovery after each GAN network is constructed.

#### Output

GPLEXUS summarizes the features of the constructed GANs, such as the subnetwork structures, the number of hub genes and functional subnetworks and the hyperlinks to constructed networks and subnetworks, which can be downloaded as delimited text files on the results page. The downloaded networks/subnetworks can be imported into the open-source Cytoscape (32) software for visualization and downstream analysis, such as Gene Ontology Set Enrichment Analysis. The user can also perform the experiment-specific condition identification analysis from the results page.

### Auxiliary tools

Auxiliary tools, such as the Gene Ontology Set Enrichment Analysis tool and the RMA-based microarray data normalization tool, have also been integrated into GPLEXUS to facilitate the use and annotation of constructed networks and subnetworks.

## RESULTS

### Comparisons between GPLEXUS and ARACNE

We compared the runtimes between the Spearman correlation-based transformation methods and ARACNE as well as the B-spline-based MI estimation method (8) using the four datasets outlined in Table 1. Our results show that the Spearman correlation-based transformation method that is implemented in GPLEXUS has a significantly reduced runtime compared with the original ARACNE method and B-spline-based MI estimation method. It was computationally infeasible to construct global GANs using large-scale genomic datasets from plant species with small genomes, such as *A. thaliana* and *G. max,* with the original ARACNE method on a typical server (DELL PowerEdge R815 Server equipped with four 8-core CPUs and 128-GB RAM) without any optimization. However, the Spearman correlation-based transformation method could compute a GAN analysis on these datasets >1000 times faster, as shown in Table 1.

Our next step was to evaluate the prediction accuracy for our methods against other methods. There were no available experimental expression datasets with known interactions in the constructed network that could be applied to test the methods. Therefore, we generated a series of synthetic gene expression datasets using the gene network modeling software, SynTReN (33). The generated expression datasets were based on experimentally validated gene interactions through the SynTRen software. The validated gene interactions for the yeast dataset have already been integrated into the SynTRen software. *A. thaliana* interaction data were downloaded from the Arabidopsis Gene Regulatory Information Server (34) and added to the SynTRen database. Different synthetic gene expression datasets with 200, 400, 600, 800 and 1000 samples for 400 yeast genes and 600 *A. thaliana* genes, respectively, were generated. We evaluated the prediction accuracy of five methods, including the original ARACNE, Spearman correlation-based transformation MI/DPI, B-Spline MI/DPI, co-expression network based on the Spearman correlation and GeneNet (35), on these benchmark datasets. The prediction accuracy was measured by the area under the receiver operating characteristic (ROC) curve (AUROC). We calculated the true positives (TP), true negatives (TN), false positives (FP) and false negatives (FN) for each method for each dataset. The ROC curve was plotted as the sensitivity [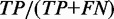] versus the 1-specificity [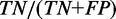]. A high AUC score indicates better performance. Our results suggest that the average AUROC score is similar across methods for the two model species (Table 2). The Spearman-based MI/DPI achieved the highest score among all of the methods that were tested. We also applied an average *F*-score [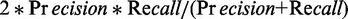] across all samples to measure the accuracy among different methods by plotting the (Precision versus Recall) PR curve as Precision 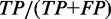 versus Recall [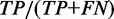]. The average *F*-score of the Spearman-based MI/DPI method is higher than the average *F*-scores of the original ARACNE method, the B-Spline MI/DPI method and GeneNet (Table 2). The average *F*-score of the Spearman MI/DPI was also significantly higher than the average *F*-score of the co-expression network. The ROC curves and PR curves for the predictions of each method for the 1000-sample expression dataset are shown in Supplementary Figures S2 and S3, respectively. The ROC and PR curves both indicate that the method used in GPLEXUS achieves an accuracy at least as high as the MI approximation methods that use a Gaussian-kernel-based method (ARACNE) or B-spline-based method, and all three methods achieve a better accuracy than the co-expression network method or GeneNet.

**Table 2.** The average performance of several integrated methods on yeast and *A. thaliana* benchmark datasets

Method	AUROC		Average F-score	
	Yeast	*Arabidopsis*	Yeast	*Arabidopsis*
Spearman-based MI/DPI	0.896	0.856	0.512	0.512
ARACNE	0.89	0.831	0.443	0.508
B-Spline-based MI/DPI	0.879	0.834	0.438	0.509
Co-expression	0.872	0.791	0.124	0.10
GeneNet	0.875	0.813	0.388	0.477

From these results, we concluded that the Spearman-based MI/DPI method can achieve a similar or better performance as the original ARACNE method and significantly accelerate the analysis runtime. Therefore, GPLEXUS enables and empowers GAN analysis for very large-scale gene expression datasets from organisms with large genomes, particularly from plants. Furthermore, this acceleration in runtime without a penalty on performance enables the powerful downstream analysis of experiment-specific condition identification.

### Construction and analysis of *A. thaliana* global GANs with GPLEXUS

We demonstrated the ability of GPLEXUS to decipher global GANs from 1848 *A. thaliana* microarray samples (Dataset II) that were measured by the Affymetrix ATH1 GeneChip, which consists of probe-sets for 22 810 genes. The network is available for download at http://plantgrn.noble.org/GPLEXUS/Result.jsp?sessionid=Arabidopsis. The global gene network includes 10 174 nodes and 161 712 edges and was constructed in <20 min using GPLEXUS with a DPI tolerance of 0.155 and MI threshold of 0.5281. Each node in the network has an average of 16 links. The constructed network could be partitioned into 154 functional subnetworks.

**Figure 1.**
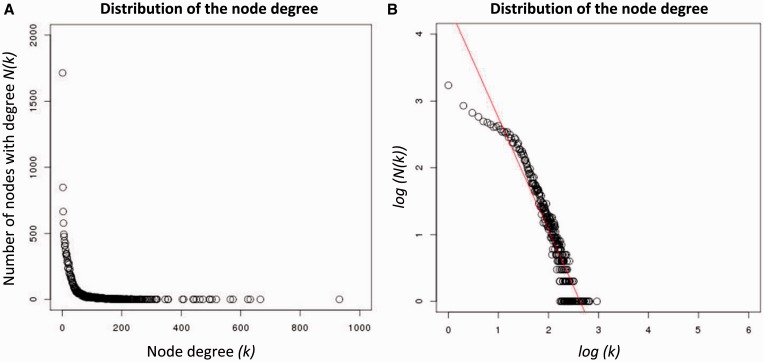
The topological properties of the constructed *Arabidopsis thaliana* gene networks. (**A**) A plot of the node degree distribution. (**B**) A plot of the node degree distribution after a log-transformation.

Many studies have indicated that the node degree distribution in biological networks follows a power-law distribution. Such networks are also known as scale-free networks (36). In a scale-free gene network, a few genes are highly connected with other genes but the majority of genes have a low number of connected genes. The network that was constructed in GPLEXUS was a typical scale-free network (Figure 1) that follows the properties of natural biological networks: most of the nodes have a few links and a few nodes have a large number of links. The degree distribution of the network followed a power-law distribution with a degree exponent of *r* = 1.67.

In a previous report (9), a global *A. thaliana* GAN with 6760 nodes and 18 625 edges was constructed using the GGM method. We focused our comparison on our constructed network with this reported study (9) to understand the properties of global gene–gene interaction networks rather than perform an exhaustive comparison between the networks, because the samples used to construct each network were significantly different. However, the networks that were derived from the GGM-based method did not exhibit the typical scale-free network property (30) [e.g. Figure 3 in (9)].

### The identification of functional subnetworks involved in diverse biotic and abiotic stress and their operating conditions in GPLEXUS

Sessile plants have evolved highly sophisticated mechanisms to survive various harsh environmental conditions, including life-threatening pathogens and pests. For example, *Botrytis cinerea* is considered the most aggressive plant fungus and causes diseases in many plant species due to its broad host range and exceptionally strong pathogenicity (37,38). The *WRKY* gene family encodes transcription factors that regulate the responses of plants to pathogen attacks and various abiotic stressors (38,39). However, little is known about the functionally associated and coordinated genes that are targets and upstream regulators of these transcription factors.

**Figure 2.**
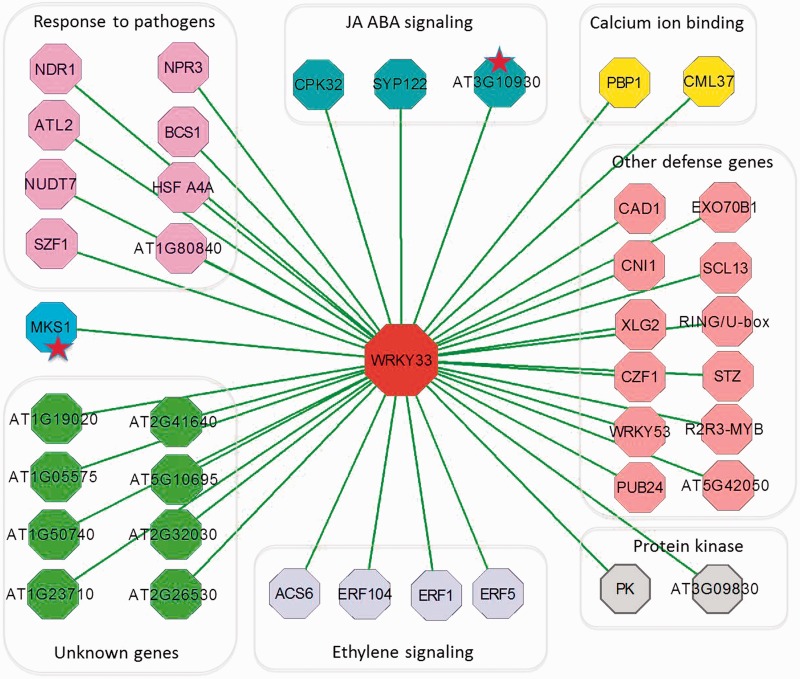
A visualization of the genes that were predicted to interact directly with the WRKY33 gene. Nodes with five-star labels are genes validated by literature.

*WRKY33* (AT2G38470) plays a critical role in the plant’s response to pathogen invasion and abiotic stress, and is known to regulate the antagonistic relationships between the defense pathways that mediate the response to pathogens (40). A total of 41 genes were associated with the *WRKY33* gene in the *A. thaliana* global GAN that we constructed (Figure 2). Of these 41 genes, 9 belong to functionally unknown genes and the remaining genes can be classified into five categories: defense pathway, ethylene (ET) pathway, jasmonate (JA) signaling pathway, abscisic acid signaling pathway and calcium ion binding. Most of these genes are involved in the defense of either biotic or abiotic stress. Plants under a pathogen attack often produce an increase in ethylene, which plays an important role in plant immunity (41). *ACS6* (AT4G11280), which is known to control the rate-liming step in ethylene biosynthesis, is involved in *B. cinerea*-induced ethylene biosynthesis, and *WRKY* acts on the pathway that induces *ACS6* expression (42,43). *ERF5* (AT5G47230) plays a positive role in the JA/ET-mediated defense against *B. cinerea* in *A. thaliana. MKS1* (AT3G18690), which encodes a nuclear substrate, is essential for basal immunity and participates in the regulation of *WRKY33* via MAP kinase 4 (*MPK4*) (44,45). *WRKY33* in turn controls the production of anti-microbial phytoalexins (46). *WRKY33* can bind to upstream sequences of genes that are involved in defense pathways that include JA signaling and ET signaling as well as camalexin biosynthesis (40,47,48). For example, the direct binding of *WRKY33* to a JA signaling gene (AT3G10930) was demonstrated in chromatin immunoprecipitation (Chip)-polymerase chain reaction experiments (47).

To identify the environmental condition(s) under which WRKY33 may interact with the 41 genes that were identified in the constructed network, we uploaded the 41 interactions to GPLEXUS and performed an experiment-specific condition analysis. The top 10 experimental conditions that were related to each gene–gene interaction were identified with *P* < 10^−6^. We checked the specific experimental conditions for some of the interactions, and almost all of the identified experimental conditions were correlated with the reported gene–gene associations. For example, *WRKY33* may induce the expression of *ACS6* under different chemical treatments. The analysis results suggest that *ACS6* may interact with *WRKY33* under salt stress (ArrayExpress accession number E-GEOD-5623, chip names GSM131321.CEL, GSM131318.CEL, GSM131322.CEL, GSM131325.CEL, GSM131329.CEL, GSM131330.CEL and GSM131317.CEL), cadmium treatment (ArrayExpress accession number E-GEOD-22114, chip names GSM549993.CEL and GSM549996.cel) and chitin treatment (ArrayExpress accession number E-GEOD-2169, chip name GSM39204.CEL). Almost all of the 41 interactions (37 of 41) are related to experiments that involve salt stress (ArrayExpress accession number E-GEOD-5623, chip name GSM131321.CEL). Therefore, these interactions suggest that the *WRKY* gene family plays an important role in biotic and abiotic stress. A review of the literature as well as of a public database (TAIR) confirms that *WRKY33* is involved in salt stress (49) and the regulation of defense pathways to various pathogens (50,51). The identified experimental conditions for all 41 interactions are described in the ‘Supplementary Materials’. This analysis confirmed that GPLEXUS can accurately capture the overall gene–gene association information and identify experiment-specific conditions from a large-scale microarray dataset.

### The identification of functional subnetworks involved in cell growth and division and their operating condition in GPLEXUS

Cell division is one of the most important and conserved biological processes related to the growth and/or development of an organism. By applying the Gene Ontology Set Enrichment Analysis to each module in the example network construction, we identified that the 22nd module/subnetwork is implicitly associated with cell division. In particular, this module is associated with the function of the *G2/M* transition of the mitotic cell cycle microtubule-based movement process. This module is shown in Supplementary Figure S4a and is similar to the pathway suggested in (52) that is shown in Supplementary Figure S4b. Most of the genes in this module were annotated with functions related to mitosis (34 genes) and cell division (56 genes) as well as microtubule cytoskeleton organization, cytoskeleton organization, organelle organization and chromosome organization. We identified an enriched mitosis-specific gene family that contains five mitosis-specific kinesin genes (AT3G20150, AT1G72250, AT2G28620, AT2G22610 and AT3G44050) and two microgametogenesis genes, *Kinesin-12A* and *Kinesin-12B* (AT4G14150 and AT3G23670, respectively) based on the Gene Ontology Set Enrichment Analysis auxiliary tool in GPLEXUS. Kinesins are a class of microtubule-associated proteins that possess a motor domain for binding to microtubules and allow movement along microtubules (53). In addition, this gene family is involved in spindle formation and chromosome movement (54).

The other gene family that was present in this module contained cyclins, which are assembled with cyclin-dependent kinases (CDKs) in the same complex to trigger the *G2/M* transition through phosphorylation (55). CCS52, a cell-cycle switch protein, works with the anaphase-promoting complex to initiate the destruction of cyclin subunits, which leads to a cell exit from mitosis. The link between the cyclin-dependent protein kinase regulators *CYCA1;1* (AT1G44110) and *CYCB1;1* (AT4G37490) was reported in (56). The link between the endocycle activator *CCS52B* (AT5G13840) (57) and the anaphase-promoting complex (*APC/C*) activating subunit *CDC20* (AT4G33260) gene was reported in (58) and the interaction between *CCS52B* and *CDKB2;2* (AT1G20930) was reported in (59). Another cyclin gene that was found in the 22nd subnetwork, *CYCD3;1* (AT4G34160), is involved in the switch from cell proliferation to the final stage of differentiation, which is a key regulatory point in the cell cycle of plants. The overexpression of *CYCD3;1* increases the length of the G2-phase and delays the activation of mitotic genes (60). *CDC25* encodes an enzyme with both arsenate reductase and phosphatase activities (52). Evidence suggests that CDC25, together with WEE1, controls the cyclin-dependent kinases that are the key regulators of cell cycle progression via phosphorylation (61).

We also performed an analysis to identify the experiment-specific conditions for all of the interactions in the 22nd module (781 gene–gene interactions). More than 200 gene–gene interactions in this module were associated with the developmental series (i.e. shoots and stems) experiments (ArrayExpress accession number E-GEOD-5633) based on the GPLEXUS analysis results. Among the 781 gene–gene interactions, 346 interactions were related to the experimental condition with chip name GSM131653.CEL, which is an experiment that focuses on the floral transition of the shoot apex before bolting. These experimental conditions are consistent with the functional annotations of this module that are related to the *G2/M* transition of the mitotic cell cycle.

### Performance comparisons between the networks constructed by GPLEXUS and ARACNE

We have demonstrated that GPLEXUS achieves a similar or better accuracy than the ARACNE method using the benchmark datasets that were generated by the SynTRen software. To further explore the efficiency of GPLEXUS compared with ARACNE, we constructed a network from Dataset II that included 10 176 genes and 169 186 edges using the ARACNE method. The constructed network can be downloaded from http://plantgrn.noble.org/GPLEXUS/Result.jsp?sessionid=Arabidopsis_gaussian_new. Even using our high-performance BioGrid system, which is used with 700 CPU cores, this network construction required >5 days with the ARACNE method.

**Figure 3.**
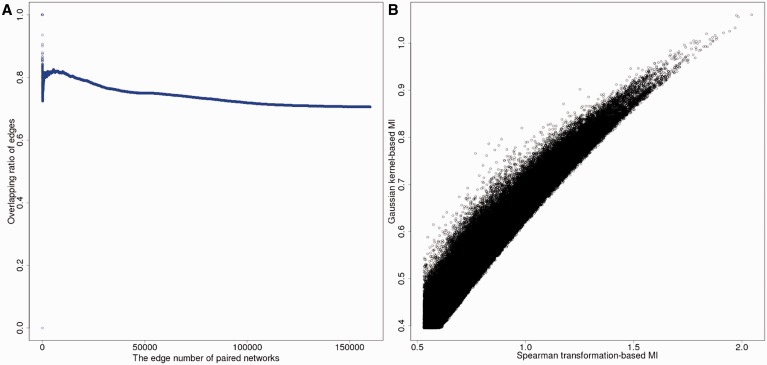
Comparisons of network edges recovered by the GPLEXUS and the ARACNE methods. (**A**) The overlap ratio of edges of the paired networks. (**B**) The distribution of the Gaussian-kernel-based MI values versus the Spearman transformation-based MI values for all overlapped edges.

We compared the overlap ratio of the networks that were constructed from ARACNE and GPLEXUS. The two networks shared 9764 genes and 117 664 edges. The statistical tests demonstrated that the overlap ratio was statistically significant with a *P*-value of 1e-30, which is far less than 0.05. The *P*-value was estimated as follows: we first randomly generated 1 million networks with the same edges and then a *t*-test was applied to estimate the statistical significance of overlap ratio. We further estimate the overlap ratio between paired networks by selecting different MI cutoff thresholds. Higher MI thresholds indicate higher probability networks with smaller number of nodes and edges. The overlap ratios of edges between paired networks under different network size are shown in Figure 3A. The overlap ratio ranged from 0.73 to 1.0. We then further compare the distribution of MI for all overlapped edges of the paired networks inferred by Gaussian kernel-based method (adopted in ARACNE) and Spearman transformation-based method (adopted in GPLEXUS),which is shown in Figure 3B. In this case, these overlapped edges with higher Gaussian kernel-based MI value in ARACNE method also have higher Spearman transformation-based MI value in GPLEXUS method. So these analyses indicate that most of the gene–gene interactions that were inferred by the ARACNE method could also be inferred by GPLEXUS.

We then compared the subnetworks that were identified by both the ARACNE method and GPLEXUS. This analysis revealed that of the top 150 subnetworks, 99 were identified by both the ARACNE method and GPLEXUS. We further checked the functional subnetworks that were inferred by GPLEXUS, including the functional subnetwork related to biotic and abiotic stress and the functional subnetwork involved in cell growth and division. The subnetwork involved in the defense against biotic and abiotic stress was identified by both methods: GPLEXUS identified 41 genes that interacted with *WRKY33* and ARACNE identified 47 genes that interacted with *WRKY33*. A total of 39 genes that were identified by GPLEXUS were also identified by the ARACNE method; however, an experimentally validated interaction between *WRKY33* and the At3g10930 gene (47) was not identified by the ARACNE method. The subnetwork involved in cell cycle division and growth that was identified by GPLEXUS was not identified by the ARACNE method.

From these results, we conclude that the performance of both GPLEXUS and the ARACNE method is similar. There exist highly overlapped genes and gene–gene interactions in the networks that were constructed by each of these methods. However, our analysis suggests that GPLEXUS is slightly more sensitive than the ARACNE method.

## DISCUSSION

Tools and methods that can be used to process and analyze large-scale genomic datasets are urgently needed, as the amount of microarray and RNA-seq data that is available in public databases accumulates. The GPLEXUS Online platform is a tool for gene network construction and analysis that significantly reduces the computational runtime compared with other methods, such as ARACNE. We demonstrated the ability of GPLEXUS to construct and analyze genome-wide GANs from both simulated and experimental data with a focus on the model plant, *A. thaliana*. We also integrated several auxiliary tools in GPLEXUS, such as the Gene Ontology Set Enrichment Analysis and RMA-based microarray data normalization tools, to facilitate the use and annotation of constructed networks and subnetworks. Our future goals are to integrate more auxiliary tools, such as network validation based on the Kyoto Encyclopedia of Genes and Genomes (KEGG) Pathway, to further improve the power of the GPLEXUS platform.

## CONCLUSION

We have developed a high-performance web-based platform for GAN construction and analysis. This system is capable of processing thousands of microarray or RNA-seq gene expression datasets from organisms with large genomes and/or a large number of genes, such as from plants, through a combination of improvements in the MI estimation method and the high-performance computing implementation and deployment. The GPLEXUS platform is as accurate and sensitive as the original ARACNE method, but produces results ∼1000 times faster. GPLEXUS also uses a condition-removing method to identify experiment-specific microarray chips from large-scale microarray datasets to gain insightful understanding of gene–gene associations. Furthermore, GPLEXUS is able to identify new functional subnetworks through the integration of the MCL. GPLEXUS Online provides interactive user interfaces to facilitate large scale gene expression data analysis and network discovery.

## AVAILABILITY

GPLEXUS is public and freely available at http://plantgrn.noble.org/GPLEXUS/. The presented case analysis input data and results are also freely available from the Web site.

The source code for the Spearman-based MI/DPI method is freely available on the GPLEXUS web server (http://plantgrn.noble.org/GPLEXUS/dataset.jsp). In addition, auxiliary tools, including tools for microarray data normalization and Gene Ontology Set Enrichment Analysis, are available under the ‘Auxiliary Tools’ menu of the GPLEXUS web server.

## SUPPLEMENTARY DATA

Supplementary Data are available at NAR Online.

## FUNDING

National Science Foundation [DBI: 0960897 to P.X.Z.]; Samuel Roberts Noble Foundation. Funding for open access charge: National Science Foundation [DBI: 0960897 to P.X.Z.]; Samuel Roberts Noble Foundation.

*Conflict of interest statement*. None declared.

## Supplementary Material

Supplementary Data
