# Supplementary material for: GPLEXUS: enabling genome-scale gene association network reconstruction and analysis for very large-scale expression data
Source: Nucleic Acids Res. 2013 Oct 30;42(5):e32. doi: 10.1093/nar/gkt983 (PMC3950724; doi:10.1093/nar/gkt983)
Supplement: Supplementary Data [file supp_42_5_e32__index.html]

GPLEXUS: enabling genome-scale gene association network reconstruction and analysis for very large-scale expression data — GPLEXUS: enabling genome-scale gene association network reconstruction and analysis for very large-scale expression data — Supplementary Data 

# GPLEXUS: enabling genome-scale gene association network reconstruction and analysis for very large-scale expression data

## Supplementary Data

files

**Files in this Data Supplement:**

- Supplementary Data - pdf file
- Supplementary Data - xlt file
