# Supplementary material for: GPLEXUS: enabling genome-scale gene association network reconstruction and analysis for very large-scale expression data
Source: Nucleic Acids Res. 2013 Oct 30;42(5):e32. doi: 10.1093/nar/gkt983 (PMC3950724; doi:10.1093/nar/gkt983)
Supplement: Supplementary Data [file supp_gkt983_nar-02617-met-n-2013-File005.pdf]

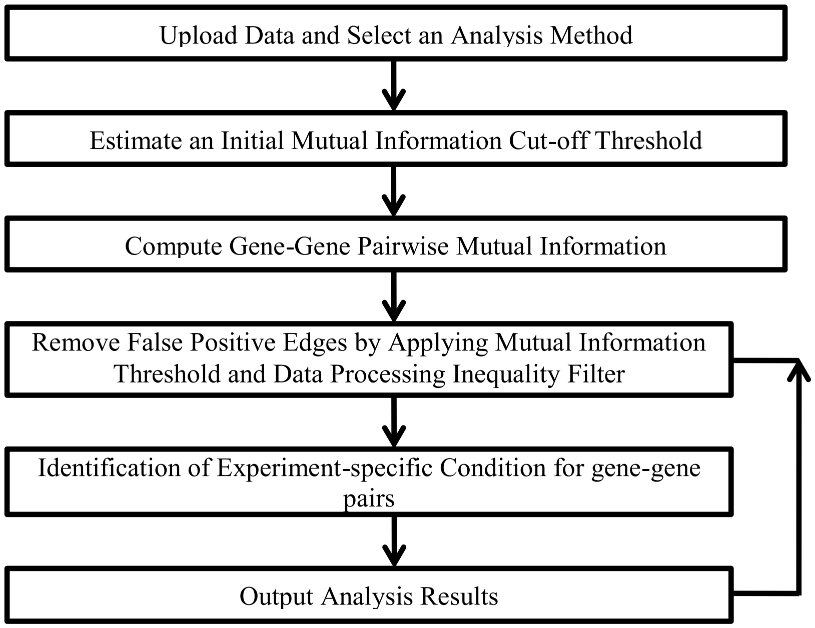

Figure S1. The GPLEXUS analysis workflow.

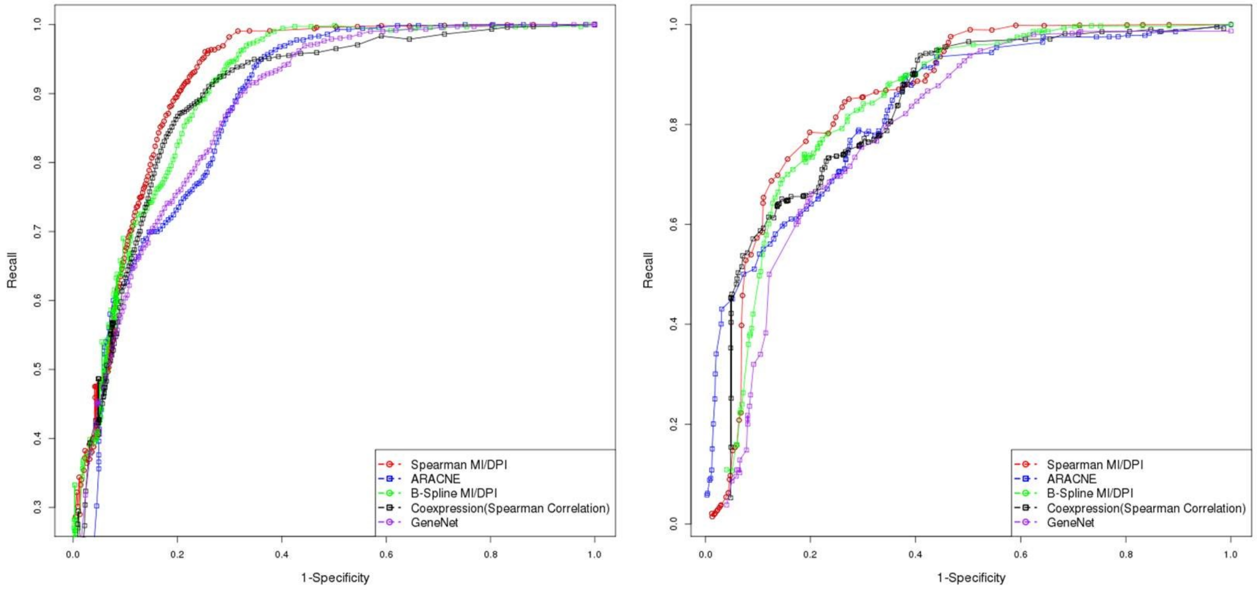

(A) (B)

Figure S2. The ROC curves for five different GAN construction methods that were applied to the synthetic gene expression datasets with 1,000 expression samples generated by the SynTRen software based on experimentally validated gene interactions in (A) yeast and (B) *Arabidopsis thaliana*, respectively. The curves were obtained by varying the MI threshold and DPI tolerance.

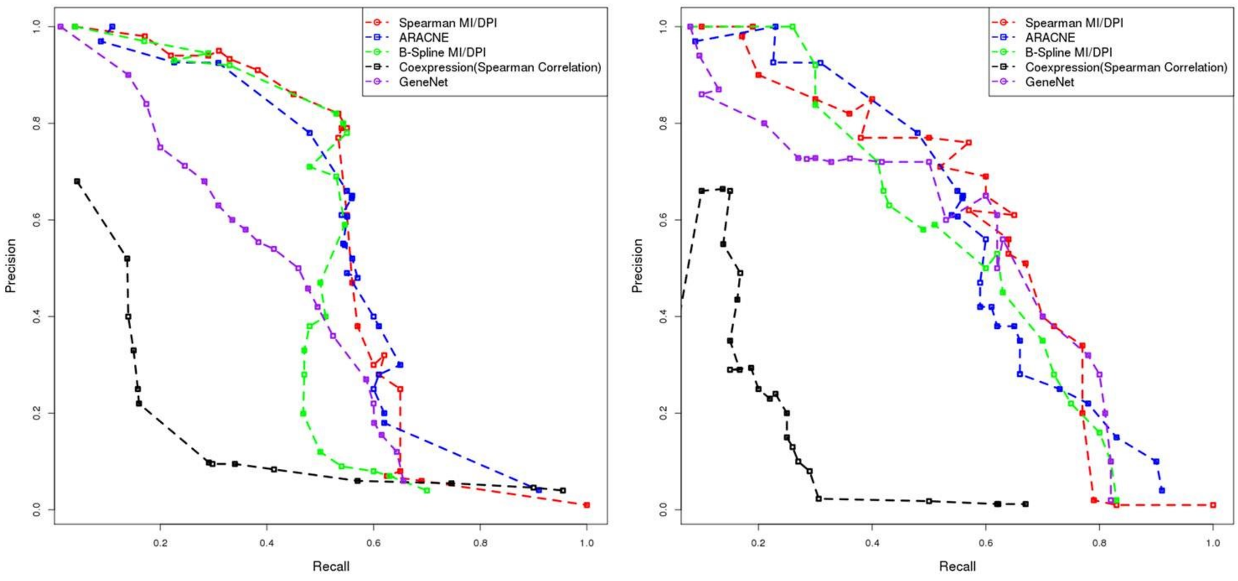

(A) (B)

Figure S3. The PR curves for five different GAN construction methods that were applied were applied to the synthetic gene expression datasets with 1,000 expression samples generated by the SynTRen software based on experimentally validated gene interactions in (A) yeast and (B) *Arabidopsis thaliana*, respectively. The curves were obtained by varying the MI threshold and DPI tolerance.

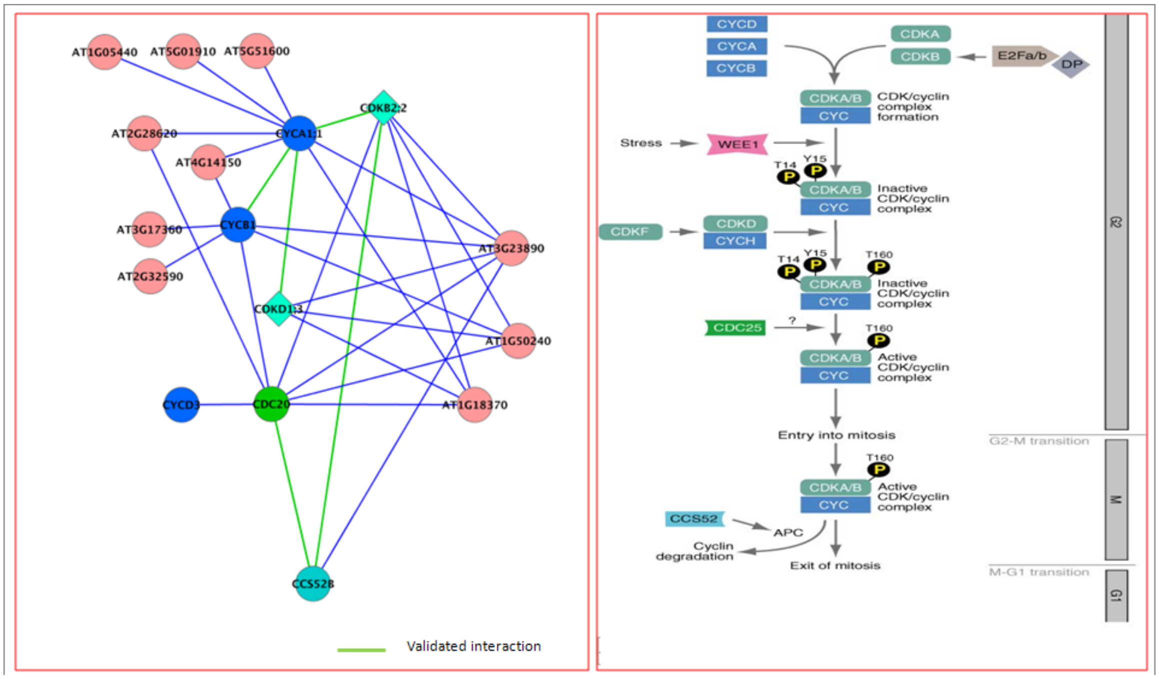

(A) (B)

Figure S4. (A) The part of the module that was identified by GPLEXUS in the *Arabidopsis thaliana* network that involves the *G2/M* transition of mitotic cell cycle microtubule-based movement. Gene-gene interactions that have been validated in the literature are highlighted in blue, cyan, and green. The link between the cyclin-dependent protein kinase regulators *CYCA1;1* (AT1G44110) and *CYCB1;1* (AT4G37490) was reported in [1]. The link between the endocycle activator *CCS52B* (AT5G13840)[2] and the anaphase-promoting complex (*APC/C*) activating subunit *CDC20* (AT4G33260) was reported in [3]. The interaction between *CCS52B* and *CDKB2;2* (AT1G20930) was reported in [4]. Validated genes are highlighted in blue, cyan, and green and predicted genes are highlighted in red. (B) A similar pathway has been suggested in [5].

References

1. Gutierrez, C., *The Arabidopsis cell division cycle.* Arabidopsis Book, 2009. 7: p. e0120.
2. de Almeida Engler, J., et al., *CCS52 and DEL1 genes are key components of the endocycle in nematode-induced feeding sites.* Plant J, 2012. 72(2): p. 185-98.
3. Kevei, Z., et al., *Conserved CDC20 cell cycle functions are carried out by two of the five isoforms in Arabidopsis thaliana.* PLoS One, 2011. 6(6): p. e20618.
4. Van Leene, J., et al., *Targeted interactomics reveals a complex core cell cycle machinery in Arabidopsis thaliana.* Mol Syst Biol, 2010. 6: p. 397.
5. Inze, D. and L. De Veylder, *Cell cycle regulation in plant development.* Annu Rev Genet, 2006. 40: p. 77-105.
